# New insights into the evasion of host innate immunity by *Mycobacterium tuberculosis*

**DOI:** 10.1038/s41423-020-0502-z

**Published:** 2020-07-29

**Authors:** Qiyao Chai, Lin Wang, Cui Hua Liu, Baoxue Ge

**Affiliations:** 1 CAS Key Laboratory of Pathogenic Microbiology and Immunology, Institute of Microbiology, Center for Biosafety Mega-Science, Chinese Academy of Sciences, 100101 Beijing, China; 2 Savaid Medical School, University of Chinese Academy of Sciences, 101408 Beijing, China; 3 Shanghai Key Lab of Tuberculosis, Shanghai Pulmonary Hospital, Tongji University School of Medicine, 200433 Shanghai, China

**Keywords:** *Mycobacterium tuberculosis*, Innate immunity, Innate immune receptors, Autophagy, Ubiquitin system, Immune evasion, Tuberculosis

## Abstract

*Mycobacterium tuberculosis* (Mtb) is an extremely successful intracellular pathogen that causes tuberculosis (TB), which remains the leading infectious cause of human death. The early interactions between Mtb and the host innate immune system largely determine the establishment of TB infection and disease development. Upon infection, host cells detect Mtb through a set of innate immune receptors and launch a range of cellular innate immune events. However, these innate defense mechanisms are extensively modulated by Mtb to avoid host immune clearance. In this review, we describe the emerging role of cytosolic nucleic acid-sensing pathways at the host**–**Mtb interface and summarize recently revealed mechanisms by which Mtb circumvents host cellular innate immune strategies such as membrane trafficking and integrity, cell death and autophagy. In addition, we discuss the newly elucidated strategies by which Mtb manipulates the host molecular regulatory machinery of innate immunity, including the intranuclear regulatory machinery, the ubiquitin system, and cellular intrinsic immune components. A better understanding of innate immune evasion mechanisms adopted by Mtb will provide new insights into TB pathogenesis and contribute to the development of more effective TB vaccines and therapies.

## Introduction

Tuberculosis (TB) remains a serious global public health threat, accounting for over 1.2 million deaths per year.^1^ *Mycobacterium tuberculosis* (Mtb), the etiological agent of TB, is estimated to have infected 1.7 billion people worldwide.^1^ Despite the availability of anti-TB medications, cure rates are low (~56% globally) for continuously emerging drug-resistant TB cases, which necessitate the use of more complex and toxic regimens and even pose risks of transmitted resistance.^1,2^ Therefore, rational design of novel TB vaccines and therapeutics based on an in-depth understanding of the intimate interplay between Mtb and host immunity is required.

Innate immunity plays a dominant role in protecting the host from early infection with Mtb, as indicated by the majority of Mtb-exposed individuals being able spontaneously control the infection despite a conspicuous delay of acquired immunity;^3^ however, an intact adaptive immune system is insufficient to restrict Mtb growth within a host deficient in innate immune responses.^4,5^ As first-line defensive patrols that quickly respond to Mtb infection, innate immune cells perform the duty of immune surveillance via a range of pattern recognition receptors (PRRs). Activation of these immune receptors leads to a range of cellular events that contribute to host anti-Mtb immunity, such as phagocytosis and apoptosis.^6^ However, long-standing coevolution with the human host protects Mtb from the effects these antibacterial mechanisms, leading to its persistent infection. Furthermore, in recently emerging pathogenic strategies, Mtb can directly target and modify various aspects of the molecular regulatory machinery of host innate immunity, such as the intranuclear regulatory machinery, the ubiquitin system and cellular intrinsic immune components, to evade host clearance. In this review, we summarize recently emerging aspects of innate immune evasion mechanisms adopted by Mtb to benefit its own intracellular survival, including the role of cytosolic nucleic acid-sensing pathways at the host–Mtb interface; novel mechanisms adopted by Mtb to circumvent host cellular innate immune events, such as membrane trafficking and integrity, cell death, and autophagy; and newly elucidated Mtb strategies to manipulate the host molecular regulatory machinery of innate immunity. A better understanding of the intricate interplay between Mtb and the host innate immune system may provide new insights into TB pathogenesis and contribute to the development of valid vaccines and therapies.

### Emerging roles of cytosolic nucleic acid-sensing pathways in host–Mtb interactions

**Fig. 1:**
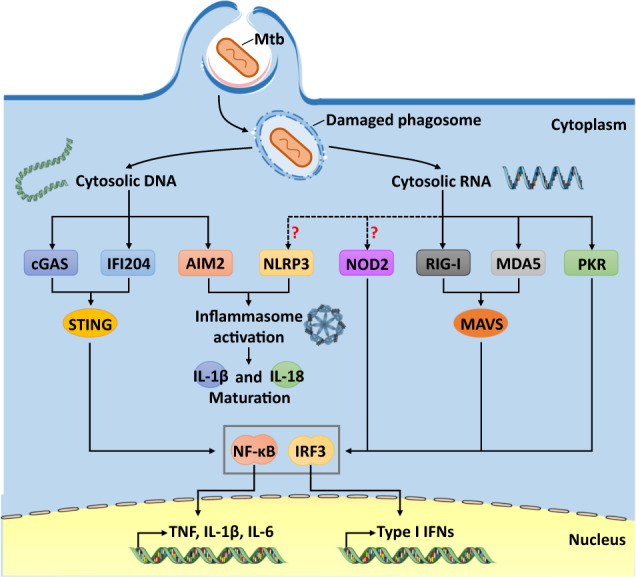
Host cytosolic DNA- and RNA-sensing pathways for the immune recognition of Mtb. Upon infection, Mtb is internalized into phagosomes by host phagocytic cells. Mtb-induced phagosome damage releases bacterial DNA and RNA into the host cytosol. The cytosolic sensors cGAS, IFI204, and AIM2 recognize Mtb DNA, while RIG-I, MDA5, and PKR detect RNA. Although NLRP3 and NOD2 also respond to Mtb infection, it remains unclear whether they are directly activated by Mtb RNA. Activated cytosolic DNA/RNA sensors further induce the activation of inflammasomes or NF-κB- and IRF3-mediated innate immune pathways to regulate host anti-Mtb responses

The core duty of the mammalian innate immune system to recognize infective pathogens is evolutionarily designed to rapidly sense and eliminate foreign threats. To prevent the successful establishment of Mtb infection in the lungs, host immune cells, and various nonclassical immune cells in the airway are equipped with a set of cell-surface and intracellular PRRs to recognize the invading mycobacteria, such as Toll-like receptors, C-type lectin receptors, Nod-like receptors (NLRs), complement receptors, and scavenger receptors (SRs). These innate immune sensors play critical roles at the interface of host mucosal immunity and Mtb pathogenesis and have been extensively reviewed elsewhere.^6–8^ In this section, we focus on the recently emerging role of cytosolic nucleic acid-sensing pathways in host–Mtb interactions (Fig. 1).

### Cytosolic DNA-sensing pathways

Although the immunostimulatory effects of mycobacterial DNA on mammalian hosts were receiving attention decades ago,^9^ host-responsive DNA-dependent cytosolic surveillance pathways were not elucidated until recently. Initially, Mtb was thought to be able to translocate from phagosomes into the cytosol by virtue of its ESAT-6 secretion system-1 (ESX-1) system during infection of host cells,^10,11^ and this process provides a potential opportunity for host cytosolic receptors to sense mycobacterial extracellular DNA. In addition, the blood of patients with active TB is characterized by a distinct transcriptional signature related to type I interferon (IFN) signaling,^12^ and this hallmark was proposed to be associated with the activation of the host cytosolic surveillance pathway, which can result in the robust production of type I IFNs.^13^ Based on these observations, Manzanillo et al. first tested the role of two putative cytosolic DNA sensors, Z-DNA binding protein 1 (ZBP1) and IFN-activable protein 204 (IFI204; the mouse ortholog of human IFI16), in host cytosolic surveillance of Mtb and found that only IFI204 contributes to the type I IFN response to Mtb infection via the stimulator of IFN genes (STING)/TANK binding kinase 1/IFN regulatory factor 3 (IRF3) axis in macrophages.^14^ Interestingly, the deletion of *Irf3* to subvert this signaling pathway in mice decreased the host expression of type I IFNs and enhanced host resistance to long-term Mtb infection.^14^ These results indicate a negative regulation of type I IFNs in host anti-Mtb immunity and suggest a potential strategy by which Mtb hijacks the cytosolic surveillance pathway to facilitate its own infection.

Cyclic GMP-AMP synthase (cGAS) is a recently characterized DNA sensor. Upon direct binding with cytosolic DNA, cGAS is activated to catalyze the production of cyclic GMP-AMP (cGAMP), leading to the activation of the downstream sensor STING.^15,16^ According to pioneering studies, cGAS functions in the cytosol, where it cooperates with STING to activate both nuclear factor-κB (NF-κB) and IRF3 signaling pathways to induce the transcription of type I IFNs and various pro-inflammatory T helper type 1 (Th1) cytokines with action against viral infections.^15–18^ Nevertheless, our recent findings and those of others suggest that cGAS can change its subcellular location and enter into the nucleus or reside on the plasma membrane, which is a possible strategy adopted by the host to distinguish self- and nonself DNA through the exertion of distinct cGAS-dependent functions.^19–21^ The involvement of the cGAS-mediated DNA-sensing pathway in host anti-Mtb immunity is indicated by the findings that cGAS expression is upregulated and that cGAS is colocalized with mycobacteria in human TB lesions, and its deficiency impairs the induction of type I IFN responses and autophagy in Mtb-infected macrophages.^22–24^ Recent studies also suggest that the cGAS/STING immune-sensing pathway is necessary for host dendritic cell (DC) activation because it increases the expression of type I IFNs upon mycobacterial infection.^25,26^ Interestingly, despite confirmation of cGAS/STING-dependent bacterial control in macrophages, *cGas*^−/−^ and *Sting*^−/−^ mice show comparable lung bacterial burden and inflammation levels to those of wild-type control mice after Mtb exposure,^22,24,25^ suggesting that additional host DNA sensors or other immune receptors may compensate for cGAS/STING-dependent antimycobacterial immune responses in vivo.

Apart from type I IFN stimulation, the detection of intracellular DNA may also lead to inflammasome activation with the production of mature pro-inflammatory cytokines, including interleukin-1β (IL−1β) and IL-18, via the absence of melanoma 2 (AIM2).^27,28^ In macrophages, AIM2 responds to Mtb genomic DNA and results in increased caspase-1 cleavage and IL-1β and IL-18 release, a finding consistent with the observation that *Aim2*-deficient mice show an increased susceptibility to Mtb infection with impaired pro-inflammatory responses.^29^ Similarly, infection with virulent *Mycobacterium bovis* can also activate the AIM2 inflammasome in macrophages.^30^ Notably, compared with nonvirulent mycobacteria containing a compromised ESX-1 secretion system, such as *Mycobacterium smegmatis*, *Mycobacterium fortuitum*, *Mycobacterium kansasii,* and attenuated Mtb H37Ra strains, virulent Mtb H37Rv has a significant inhibitory effect on AIM2-dependent innate cytokine responses.^31^ This finding seemingly contradicts the accepted idea that ESX-1 is essential for activating host cytosolic surveillance pathways. Most likely, ESX-1 is required for Mtb to deliver a number of effectors into the host to remodel the intracellular environment to improve its chance for survival, despite its role in inducing immune recognition. In addition, it should be noted that individual effectors delivered by the Mtb ESX-1 secretion system may play independent immunoregulatory roles with different host targets, and thus, the mechanisms underlying ESX-1-dependent stimulation or evasion of the host cytosolic surveillance pathway both function during host–Mtb interactions. This notion is supported by the finding that blocking the secretion of EsxA, a major substrate of ESX-1, significantly reduced cGAS/STING-mediated IFN production while leaving the inflammasome-mediated IL-1β response virtually intact.^23^ Furthermore, there is intimate cross talk between components of the AIM2 inflammasome and the cytosolic cGAS/STING-sensing pathways during Mtb infection, as indicated by a recent study indicating that apoptosis-associated speck-like protein, a key adapter that mediates the downstream signaling pathways of AIM2 inflammasomes, interacted with STING and negatively regulated the host type I IFN response to Mtb infection.^32^ Therefore, specifically targeting mycobacterial ESX-1 products or host regulatory factors might enable the selective regulation of inflammasome and cGAS/STING pathway activation and, hence, contribute to the recovery of the equilibrium between Th1-type cytokine and type I IFN responses in TB patients to improve their anti-Mtb immunity.

### Cytosolic RNA-sensing pathways

The immunomodulatory activity of mycobacterial RNA in mammalian hosts received attention as early as the 1960s and 1970s.^33^ Recently, it was reported that Mtb-infected macrophages can deliver extracellular vesicles (exosomes) containing abundant mycobacterial RNA to recipient cells, suggesting that Mtb RNA is probably released into host cells to trigger the RNA-dependent cytosolic surveillance pathway.^34^ The cytosolic RNA-sensing pathway was initially identified as a key part of host immune surveillance against RNA virus infection. In mammalian cells, retinoid acid-inducible gene I (RIG-I)-like receptors (RLRs) are well-conserved cytosolic PRRs that recognize cytosolic viral RNAs and activate downstream immune pathways to promote the production of type I IFNs and other pro-inflammatory cytokines.^35^ RIG-I and melanoma differentiation-associated protein 5 (MDA5) are the best characterized RLRs, which preferentially recognize short polyphosphorylated double-stranded RNA (dsRNA) and long dsRNA, respectively.^36^ After sensing foreign RNAs, RIG-I, and MDA5 transmit signals via a common adapter, mitochondrial antiviral signaling (MAVS), which forms large prion-like polymers and recruits tumor necrosis factor (TNF) receptor-associated factors (TRAFs) to further activate NF-κB and IRF3 immune signaling pathways.^37–39^ Recently, the RLR-mediated cytosolic surveillance pathway was also shown to participate in the host immune response to various bacterial pathogens, such as Mtb, *Legionella pneumophila*, *Helicobacter pylori,* and *Listeria monocytogenes*.^40–42^ The involvement of the RLR-dependent RNA-sensing pathway during host–Mtb interactions is implied by the increased expression of RIG-I and MDA5 mRNAs in Mtb-infected macrophages.^43^ Further investigation of recombinant Mtb strains demonstrated that Mtb SecA2 and ESX-1 secretion systems are critical for the delivery of Mtb RNA into the host cell cytosol, resulting in IFN-β production through the host RIG-I/MAVS-mediated RNA-sensing pathway.^44^ The role of the MDA5-mediated RNA-sensing pathway in detecting Mtb infection was also confirmed by a recent study, which showed that deletion of MDA5 impaired IFN-β production and bacterial control in human macrophages, results similar to those obtained by the deletion of RIG-I or MAVS.^45^ Nonetheless, RIG-I, not MDA5, appears to interact with the Mtb-specific mRNAs *polA* and *ppe11* (ref. ^44^), suggesting that these RLRs probably play nonredundant roles in detecting different types of mycobacterial RNAs. Furthermore, *Mavs*-deficient mice showed obviously increased resistance to Mtb infection with attenuated bacterial growth in their lungs,^44^ as was also observed in *Irf3*-deficient mice,^14^ supporting a potentially negative role of type I IFNs in host anti-Mtb immunity in vivo.

In addition to the RIG-I/MDA5/MAVS axis, protein kinase R (PKR) has been identified as another host sensor of cytosolic dsRNA, which can interact with the natural RNA derived from diverse viruses or bacteria,^45^ leading to the activation of IRF3, NF-κB, and other various innate immune signaling pathways.^46^ According to an infection model based on the interaction of *M. bovis* bacillus Calmette–Guérin (BCG) and primary human blood monocytes, the mycobacteria-induced production of inflammatory cytokines is regulated by the phosphorylation and activation of PKR.^47^ A recent study also demonstrated that Mtb infection results in +increased expression of PKR and increased phosphorylation of its substrate, eukaryotic translation initiation factor 2 A, in human cells, and PKR deficiency leads to enhanced intracellular growth of mycobacteria.^48^ However, the in vivo role of PKR in host immunity challenged by Mtb infection remains unclear. Although a research group has reported that mice lacking PKR show reduced mycobacterial burden with less severe pulmonary pathology than shown by wild-type mice,^49^ they recently attributed this observation to different genetic backgrounds of the mice rather than to a direct role of PKR.^50^

Aside from the RIG-I/MDA5- and PKR-mediated cytosolic RNA-sensing pathways, intracellular NLR family members, including NLRP3 and NOD2, can also recognize foreign dsRNA and single-stranded RNA (ssRNA), respectively.^51,52^ Mtb infection activates both of these NLRs in an ESX-1-dependent manner to trigger various host downstream innate immune responses, such as NLRP3 inflammasome formation, autophagy initiation and NF-κB and IRF3 pathway activation, which have been extensively reviewed elsewhere.^6,52^ However, it is still unclear whether NLRP3 and NOD2, which respond to a range of pathogen-derived stimuli,^52,53^ can be activated by direct binding to mycobacterial extracellular RNAs, although a recent study reported that dsRNA from Mtb cultures is able to induce caspase-1 activation in retinal pigment epithelium.^54^

In summary, host cytosolic DNA- and RNA-sensing pathways are newly emerging innate immune recognition mechanisms of host–Mtb interactions. Growing evidence indicates that there is intimate cross talk among the components of different cytosolic nuclear acid-sensing pathways,^23,44^ and these immune surveillance pathways probably play nonredundant roles in host anti-Mtb immunity. However, the in vivo data from animal infection models show that activation of cytosolic cGAS- or RLR-mediated sensing pathways can induce a strong type I IFN response that appears to impair host resistance to mycobacterial infection,^14,44^ suggesting that Mtb may exploit the host cytosolic surveillance pathways to facilitate its own growth. In contrast, activation of other cytosolic pathways during Mtb infection, such as that mediated by AIM2, NOD2, and NLRP3, can promote the production of protective inflammatory cytokines. Hence, further investigation may be focused on how to spatiotemporally and selectively regulate these cytosolic surveillance pathways to optimize host anti-Mtb immunity. Furthermore, a recent study demonstrated that drug treatment targeting cytosolic RNA sensors benefited the host by controlling mycobacterial intracellular growth,^48^ highlighting the potential value of targeting the cytosolic immune surveillance pathway for novel host-directed anti-TB therapy.

### New aspects of Mtb-modulated cellular innate immune events

**Fig. 2:**
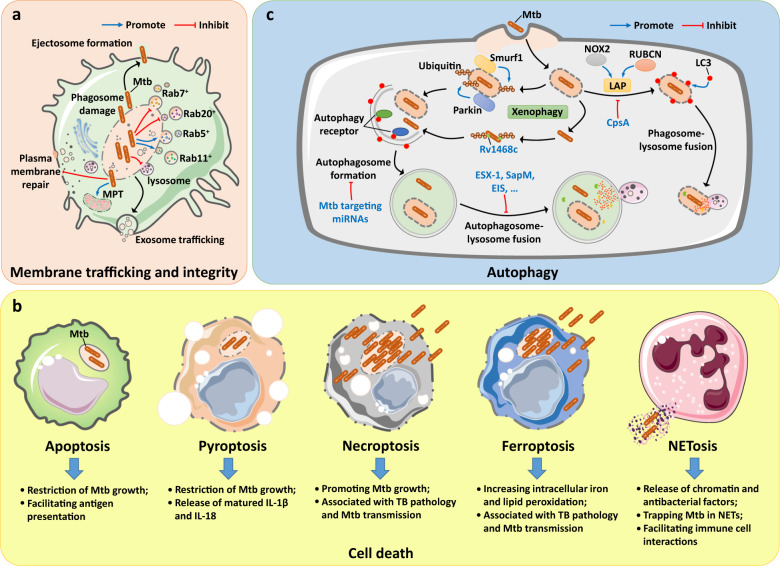
Host cellular innate immune events during Mtb infection. **a** Mtb regulates cellular membrane trafficking and integrity. Mtb permits Rab5^+^ and Rab11^+^ but restricts Rab7^+^ and Rab20^+^ endosome recruitment to the phagosome. In addition, Mtb prevents phagosome–lysosome fusion and causes phagosome damage enabling its escape into the host cytosol. Furthermore, Mtb induces mitochondrial membrane permeability transition (MPT), inhibits host cell plasma membrane repair, and regulates ejectosome formation and exosome trafficking. **b** Host cell death modalities upon Mtb infection. Diverse cell death modes may be induced during Mtb infection, and of these types, apoptosis and pyroptosis are proposed to restrict bacterial intracellular growth and facilitate anti-Mtb immune responses, whereas necroptosis and ferroptosis benefit Mtb replication and transmission. In addition, neutrophil extracellular trap (NET)-associated NETosis likely traps Mtb in NETs and facilitates immune cell interactions. **c** Mtb modulates the host autophagy process. Both the xenophagy and LC3-associated phagocytosis (LAP) pathways contribute to host autophagy-related immune clearance of Mtb. The anti-Mtb xenophagy pathway is alternatively mediated by the E3 ubiquitin ligases Parkin- and Smurf1-dependent ubiquitination of Mtb and Mtb phagosomes or by direct binding of ubiquitin to Mtb surface Rv1468c; the LAP pathway is mediated by NOX2 and RUBCN, which promote LC3 recruitment on Mtb phagosomes. However, Mtb could disrupt both xenophagy and LAP pathways by modulating host miRNAs or by employing a range of protein effectors, such as ESX-1, SapM, EIS, and CpsA

The activation of host innate immune-sensing pathways by Mtb infection leads to a range of subsequent cellular antimicrobial events, such as phagocytosis and apoptosis; however, these effects can be modulated by Mtb to benefit its long-term intracellular survival.^6^ In this section, we focus on recently emerging aspects of regulatory strategies adopted by Mtb to interfere with host membrane trafficking and integrity, cell death, and autophagy processes (Fig. 2).

### The manipulation of membrane trafficking and integrity by Mtb

The leveraging of host membrane trafficking in infected cells is a key strategy for the notorious success of Mtb as a highly adapted intracellular pathogen. Upon infection, Mtb is engulfed by host phagocytic cells such as macrophages, neutrophils, and DCs and internalized in a phagosome, the organelle responsible for routine clearance of pathogens. Notably, while phagosomes in macrophages and neutrophils are generally designated to rapidly eliminate pathogen-associated cargo, DC phagosomes tend to moderately degrade their internalized substrates to preserve antigenic peptides for priming adaptive immune responses.^55^ However, it has been well documented that Mtb recruits the GTPase Rab5, but not Rab7, away from the phagosome to inhibit phagolysosome maturation.^56,57^ The prevention of the biogenesis of phagolysosomes plays a vital role in Mtb infection, transmission, latency, and immune evasion.^56,57^ Multiple routes and numerous effectors are employed by Mtb for the suppression of phagosome maturation and acidification, which have been extensively summarized elsewhere.^6,57^ Notably, the ability of Mtb to manipulate host membrane trafficking may also contribute to the targeting of the host endosomal sorting pathway by human immunodeficiency virus during viral budding, thus favoring synergism of these two pathogens during coinfection.^58^

Recently, the spatiotemporal dynamics of Mtb phagosomal morphology and composition have received growing attention. During maturation, phagosomes associate with early and late endosomes, as well as other intracellular organelles such as Golgi-derived vesicles, the endoplasmic reticulum (ER) and mitochondria,^59^ and these interactions are very dynamic and can promptly change both the phagosomal membrane and luminal components with the principal aim of restraining the growth of internalized pathogens. Upon infection, Mtb alternatively localizes to two morphologically different types of phagosomes, tight and spacious phagosomes, which are consistently observed in both TB patients and other animal hosts.^60–62^ A recent study revealed that IFN-γ can facilitate endosomal interactions with Mtb phagosomes via the regulation of the Rab20-dependent vesicular trafficking pathway, which promotes membrane influx into tight phagosomes and shifts them into spacious and proteolytic compartments that restrict Mtb growth.^63^ However, Mtb can avoid being directed to Rab20-positive spacious phagosomes via its ESX-1 system. Another study has demonstrated that patient-derived Mtb strains can produce large amounts of 1-tuberculosinyladenosine (1-TbAd), which acts as a bacterial antacid and selectively accumulates in host cellular acidic compartments, resulting in phagosomal swelling and the obliteration of the lysosomal multilamellar structure.^64^ The phagosomal components also appear to be fine-tuned by mycobacteria, given that the Mtb-specific phagosome proteome shows distinct characteristics from that of latex bead- or other bacterial pathogen-containing phagosomes.^65^ It is conceivable that Mtb must remodel the intravacuolar microenvironment to establish a pathogen-friendly niche. For example, Mtb can encode various effectors, such as PtpA, 1-TbAd, and MarP, to elude, neutralize or tolerate the acidic environment of phagosomes.^64,66,67^ Mycobacteria also avoid being trafficked with bactericidal molecules, such as lipocalin 2, an innate immune protein that disrupts bacterial iron acquisition, to their compartments while retaining access to transferrin for iron uptake through the Rab11^+^ endocytic recycling pathway.^68^ The change in Mtb phagosomal content is also a hallmark of accumulated lipid droplets, which probably depends on Rab7, according to a recent study.^69^ Although it was proposed that Mtb can disrupt mitochondrial fatty acid oxidation to promote lipid body deposition in macrophages for utilization,^70^ another study demonstrated that increased formation of lipid droplets in Mtb-infected cells actually facilitates host biosynthesis of eicosanoids and restricts bacterial growth.^71^ Therefore, the multifaceted role of lipid bodies in Mtb phagosomes requires further elucidation.

Membrane rupture, which depends on the mycobacterial ESX-1 system, is another typical characteristic of Mtb phagosomes.^10,11,72^ This phenomenon has long been considered a pathogen-driven event utilized by Mtb to escape from a bactericidal phagosome and enter the host cell cytosol, where it can obtain abundant nutrients. However, a recent study demonstrated that the inhibition of phagosomal maturation and acidification is a precondition for Mtb phagosomal damage.^72^ Furthermore, as identified in other successful intracellular pathogens, such as *L. pneumophila* and *Brucella abortus*, the establishment of a sheltered niche within a vacuolar compartment mimics a normal cellular organelle and enables the pathogen to avoid host immune surveillance and clearance.^73,74^ This finding one to wonder why a mycobacteria departs from a cozy niche to enter the cytosol where it must confront a series of cytosolic immune sensors? To date, no direct evidence indicates an obvious advantage of mycobacterial extra-phagosomal survival. One possible explanation is that the success of persistent Mtb infection requires the ESX-1 secretion system to damage the phagosomal membrane and deliver numerous secretory effectors into the cytosol to target and regulate cellular immune components. This assumption is supported by accumulating evidence that indicates an indispensable role for the ESX-1 system in Mtb pathogenesis, as it has been linked to host cytosolic surveillance evasion,^23,31^ phagosome maturation arrest,^63,75^ cell death reprogramming,^76,77^ autophagy inhibition,^78,79^ etc. Alternatively, escape from phagosomes facilitates Mtb ESX-1-dependent plasma membrane damage, facilitating efficient Mtb replication and spread to neighboring cells and, eventually, to new hosts. By using time-lapse microscopy at the single-cell level, ruptured host cell plasma membranes were observed at the contact points of Mtb with the plasma membrane, which provides direct evidence for this assumption.^80^ However, this evidence does not exclude the other possibility: the host may actively promote Mtb phagosome rupture at the early stage of infection to eliminate the pathogen. As described above, host cytosolic immune sensors,^22–24,29,43,44,48,51,52^ as well as other diverse defense molecules,^63,81,82^ can recognize and target either damaged Mtb phagosomes or cytosolic mycobacteria for immune clearance. Accordingly, a recent work revealed that a host deficient in endosomal sorting complex required for transport, machinery thought to be important for repairing ESX-1-dependent damage of mycobacteria-containing vacuoles, shows restricted intracellular bacterial growth.^83^ In addition, several independent studies using different experimental methods consistently found that the majority of intracellular mycobacteria are not localized in the host cytosol until a very late stage of infection,^10,11,72^ suggesting that Mtb may prepensely escape from phagosomes for rapid replication and preparation for further transmission, which occurs only after the host cells are compromised by immune responses that are attenuated after prolonged interaction with the mycobacteria.

Aside from membrane changes related to phagosome–lysosome fusion (and autophagosome formation, which is discussed below), recent studies have indicated that Mtb is also involved in the modulation of other cellular membranes. For example, the translocation of the Golgi apparatus and lysosome-derived vesicles to the plasma membrane is required for the repair of mycobacteria-induced disruptions of the macrophage plasma membrane, whereas virulent Mtb strains are able to prevent this process and induce necrosis of infected cells.^76^ In addition, Mtb infection has also been associated with the induction of mitochondrial membrane permeability transition (MPT), which causes host cell necrosis.^84–86^ Interestingly, pathogenic mycobacteria may also coopt the host autophagic machinery to break through the plasma membrane and depart from their host cells through an F-actin-based vacuolar compartment termed an “ejectosome”, which is proposed to be a nonlytic cell-to-cell bacterial transmission mechanism.^87,88^ Furthermore, Mtb can alter the protein composition of exosomes secreted by infected human macrophages.^89^ These actions indicate that Mtb is involved in the host exosome-related vesicular trafficking pathway, but its significance for TB pathogenesis remains largely unexplored. In conclusion, the success of the intracellular lifestyle of Mtb largely depends on the establishment of an easeful niche within a nonfusogenic phagosome. In fact, growing evidence suggests that the phagosome is more likely serving as a signaling platform than as clearance machinery,^90^ and Mtb is likely to promptly interact with the cellular membrane trafficking system to sense and change the host immune and metabolic conditions. These assumptions, as well as the potential interplay between Mtb and other host cellular organelle membranes, warrant further in-depth investigations.

### Reprogramming cell death by Mtb

The development of central necrosis in granulomatous lesions, which induces lung cavity formation and promotes Mtb transmission to another human host, is a hallmark characteristic of severe TB cases.^91^ Hence, Mtb-induced host cell death during infection likely plays a crucial role in TB pathogenesis.

Initially, virulent Mtb strains were thought to induce host cell apoptosis in an ESX-1-dependent manner, as indicated by an in vitro infection model using immortalized murine macrophage cell lines.^92–94^ However, several studies using human macrophage cell lines have indicated that virulent Mtb leads to a lower apoptosis rate than attenuated strains^95–97^ and even inhibits apoptosis by employing a wide variety of effector proteins (which are effectively summarized in ref. ^98^) to evade host downstream immune responses. Most likely, the integrity of cell death-associated molecular pathways in certain cell lines accounts for these discrepancies. Further investigations suggested that virulent Mtb strains can switch the induction of host cell apoptosis to necrosis via manipulation of eicosanoid metabolism pathways.^76,77^ In contrast to apoptosis, which is proposed to result in the containment of mycobacteria,^98^ the propensity of Mtb for inducing necrotic death likely benefits the release of bacteria into the permissive extracellular microenvironment they have modulated for better growth.^99^ However, a recent study using time-lapse imaging suggested that Mtb-induced necrosis predominantly benefits the growth of the bacteria within dead cells, as indicated by the observation of the accelerated intracellular replication of Mtb after host macrophage death, which was much faster than it was in either live cells or in the extracellular milieu.^100^ In addition, the phagocytosis of dead infected cells containing aggregated mycobacteria by bystander macrophages would cause further necrosis.^100^ Regardless of the debate on the benefit of necrosis on intra- or extracellular mycobacterial growth, these studies have established the currently accepted concept suggesting that Mtb can reprogram host cell death and that it preferentially induces necrosis rather than apoptosis to facilitate its survival and dissemination.

More recent studies have pointed out that mycobacteria-induced host cell necrosis is a programmed cell death process, termed “necroptosis”, which is stimulated by host TNF via TNF receptor 1 (TNFR1) and is dependent on receptor-interacting serine-threonine kinases 1 (RIPK1)/RIPK3.^84,85^ Mtb infection markedly increases mixed-lineage kinase domain-like protein (MLKL), the effector protein in the RIPK1/RIPK3-mediated necroptosis pathway, and other pronecroptotic molecules such as TNFR1 and ZBP1 (ref. ^84,101^). However, deletion of MLKL or inhibition of RIPK1 in macrophages does not completely rescue Mtb-infected cells from death,^84,101^ suggesting that, although the deficiency of MLKL or RIPK1 can abrogate the canonical necroptosis pathway, upstream TNF/TNFR1-mediated signaling may stimulate the induction of other cell death pathways during Mtb infection. Alternatively, Mtb may bypass the TNF/TNFR1/RIPK1 cascade to cause necroptosis, a notion supported by a recent study showing that Mtb can secrete a nicotinamide adenine dinucleotide (NAD^+^) glycohydrolase to induce host cell necroptosis independent of RIPK1 and TNF.^102^ Furthermore, MLKL-deficient or RIPK1-inhibited humanized mice exhibited bacterial burdens and lung histopathology indistinguishable from those of the control mice in response to Mtb infection.^101^ These results imply that, although TNF/TNFR1/RIPK1-dependent necroptosis is activated by Mtb, this type of cell death seems to play a restricted role in TB pathogenesis. Hence, additional mechanisms underlying Mtb-induced host cell death and their association with TB pathogenesis should be taken into account.

In addition to those identifying necroptosis, a number of studies have identified multiple other types of programmed necrosis in mammalian host cells in response to Mtb infection, such as inflammasome-mediated pyroptosis and neutrophil extracellular trap (NET)-associated NETosis, which have recently been extensively reviewed.^98^ Notably, it was reported that Mtb inhibited macrophage inflammasome activation and pyroptosis via its secreted effectors Zmp1 and Rv3364c, thus limiting host pro-inflammatory immune responses.^103,104^ Furthermore, NETosis seemingly facilitates the interactions between neutrophils and other immune cells rather than killing Mtb directly.^105,106^ More recently, Amaral et al. found that Mtb-induced macrophage necrosis was characterized by elevated levels of intracellular iron and mitochondrial superoxide. increased lipid peroxidation, and downregulated glutathione and glutathione peroxidase-4, findings that are in line with the hallmark characteristics of a typical and regulated necrosis process termed “ferroptosis”.^107^ Using a mouse model of acute Mtb infection, the same group confirmed the association between lung necrosis and Mtb-induced ferroptosis, which indicated that ferroptosis probably contributes to TB pathology and allows Mtb to thrive and spread.^107,108^ More importantly, treatment with the ferroptosis inhibitor ferrostatin-1 reduced the bacterial burdens and attenuated pulmonary necrosis in acutely Mtb-infected mice,^107^ suggesting that the targeting of the host ferroptotic pathway may be a potential strategy to control TB infection and reduce lung damage.

In summary, diverse host cell death pathways are involved in Mtb infection, acting either as host protective mechanisms or as bacterial survival strategies. Notably, the preference for these different cell death modalities likely depends on both the mycobacterial strains and molecular integrity of cell death pathways in a certain host cell type. Therefore, identification of and interference with mycobacterial effectors or potential host molecular switches that can control the death modes of infected cells might be a new approach to control TB infection and diminish Mtb-caused tissue damage.

### Exploitation of the autophagy process by Mtb

Our knowledge of the physiological and immunological roles of autophagy has recently expanded greatly.^109^ Autophagy is a cellular mechanism evolutionarily conserved from yeast to mammals that involves the degradation of cellular materials such as damaged organelles, unwanted proteins or foreign pathogens by capturing them in a double-membrane structure termed the “phagophore”, which can subsequently develop into a mature autophagosome and fuse with lysosomes.^109,110^ The protective role of autophagy in host defense against Mtb was first investigated by Gutierrez et al., who noted that a portion of mycobacteria are sequestered into autophagosome-like compartments during infection in macrophages and that exogenous stimulation to enhance autophagy restricted Mtb intracellular survival.^111^ A subsequent study confirmed this observation and revealed that while the Th1 cytokine IFN-γ can induce host macrophage autophagy to control Mtb infection, the Th2 cytokines IL-4 and IL-13 abrogate such autophagy-mediated killing of intracellular mycobacteria.^112^ Furthermore, it has been reported that autophagy is involved in regulating other multiple anti-Mtb mechanisms, such as the mycobactericidal capacity of the lysosomal soluble fraction,^113^ the expression of SRs on macrophages,^114^ and mycobacterial antigen presentation.^115^ Taken together, these findings indicate an essential role of autophagy in both host innate and adaptive immunity in Mtb infection.

More recently, researchers noted that eukaryotic cells could allocate specific cellular materials to the autophagy pathway, which is considered a selective process. Host selective autophagy of foreign pathogens is termed “xenophagy”.^109^ Deletion of xenophagy-associated genes leads to significantly enhanced mycobacterial survival in macrophages and in mice,^22,24,81,116–120^ further supporting a protective role of autophagy in host anti-Mtb immunity. During Mtb infection, ubiquitin-ligating (E3) enzyme-mediated ubiquitin attachment to bacteria is a key step for host initiation of xenophagy, through which various autophagy receptors, such as p62 (SQSTM1), NBR1, NDP52, and optineurin, are recruited and subsequently engage with autophagosomal membrane-associated protein LC3 to capture bacteria into autophagosomes.^81,116–120^ To date, only two E3 ubiquitin ligases, Parkin and Smurf1, have been found to control ubiquitin targeting of Mtb for xenophagy initiation, which was realized through the mediation of K63- and K48-linked ubiquitination of Mtb-associated substrates, respectively.^118,119^ In addition, a recent study demonstrated that human makorin ring finger protein 1 (MKRN1) is an Mtb-specific E3 ubiquitin ligase that can mediate the ubiquitination of Mtb in vitro in conjunction with ubiquitin-activating enzyme E1 (UBE1) and ubiquitin conjugating enzyme E2 D3 (UBE2D3),^121^ although its intracellular role during Mtb infection has not been illustrated. However, the protein substrates on Mtb-containing phagosomes or mycobacterial surfaces that can be ubiquitinated by these E3 ligases remain unidentified. *Parkin*^−/−^ mice fail to restrict Mtb replication during acute infection, and *Smurf*^−/−^ mice display an attenuated capacity to control Mtb infection during the chronic phase,^118,119^ suggesting that they have different roles in host anti-Mtb immunity. Apart from E3 ligase-mediated xenophagy, we recently identified an Mtb surface protein, Rv1468c, which can directly bind host cytosolic ubiquitin chains via a eukaryotic-like ubiquitin-associated (UBA) domain to recruit autophagy components and trigger a xenophagic response.^81^ Therefore, both E3 ligase-dependent and E3 ligase-independent mechanisms are involved in host ubiquitin targeting of intracellular Mtb for xenophagy initiation. Furthermore, it is notable that the host can also drive ubiquitin-independent xenophagy. In *Salmonella typhimurium*-infected cells, host galectin-8 detects invading bacteria by binding glycans on damaged bacteria-containing vacuoles and further interacts with the autophagy receptor NDP52 to recruit LC3 and activate antibacterial autophagy.^122^ Given that galectins also participate in the cytosolic recognition of Mtb-damaged phagosomes,^63,82^ ubiquitin-independent xenophagy may also occur during Mtb infection. In addition, in view of growing eukaryotic-like effectors identified in Mtb,^123,124^ it is not surprising that Mtb might retain certain surface proteins that can be directly recognized by autophagy receptors or LC3 family proteins via protein–protein interaction motifs to trigger host xenophagy.

In response, Mtb adopts multiple strategies to avoid autophagy-related immune clearance during infection, and an effective mechanism involves directly or indirectly targeting autophagy machinery by delivering effector proteins into host cells. For example, Mtb-secreted acid phosphatase (SapM) has been found to target host Rab7 to prevent autophagosome–lysosome fusion.^123^ Another Mtb effector, enhanced intracellular survival (EIS), which is an *N*-acetyltransferase that has been reported to increase the acetylation level of histone H3 to upregulate IL-10, results in autophagy suppression via the activation of the Akt/mTOR/p70S6K pathway.^125^ Recently, a host noncanonical autophagy pathway, named LC3-associated phagocytosis (LAP), was identified in the context of a fungal infection and involved in the recruitment of LC3 and other components of the canonical autophagy machinery on pathogen-containing phagosomes for lysosomal degradation.^126^ Notably, LAP does not rely the preinitiation complex in ULK1 signaling, instead requiring Rubicon and NADPH oxidase 2 (NOX2), molecules, which are not involved in the canonical autophagy pathway.^126^ Interestingly, Mtb is insensitive to NADPH oxidase and LAP trafficking, and *Nox2*-deficient mice show few differences compared with the control mice in controlling Mtb infection.^127^ The Mtb protein CpsA has been proven to cause autophagy resistance,^127^ but its direct target in the host LAP pathway remains unclear. Interference with host microRNAs (miRNAs) is another efficient strategy by which Mtb disturbs the host autophagy pathway, as shown by miRNA often simultaneously targeting multiple interrelated genes, thereby leading to a potent cumulative effect on a certain molecular pathway.^128^ Mycobacteria can modulate the expression of diverse host miRNAs, such as miR-33 and its passenger strands miR-33*, miR‐125a, miR-17, miR-155, and MIR144*, which results in autophagy inhibition through the direct repression of a wide range of key autophagy effectors.^70,129–132^ In addition, we recently found that Mtb infection induces the expression of miR-27a, the miRNA that targets the ER-located Ca^2+^ transporter CACNA2D3 to inhibit the downstream calcium-associated xenophagy pathway in the host.^133^ Taken together, these findings support a prevailing view that autophagy is a host mechanism of intrinsic defense against intracellular bacteria, and under certain circumstances, Mtb attempts to adopt it for its own benefit.

Several recent studies have raised questions about the exact role of autophagy in host–Mtb interactions. On the one hand, growing studies support an autophagy-independent role of the autophagy machinery during infection.^134^ For example, a study showed that mice lacking *Atg3*, *Atg7*, *Atg12*, *Atg14,* or *Atg16l1* in myeloid cells displayed few differences in bacterial loads compared with those of the control mice during acute Mtb infection and argued that host *Atg5*-dependent resistance to Mtb predominantly depends on its regulatory functions in neutrophil-related immunopathology rather its function in the autophagy pathway.^135^ Hence, the multifaceted protective role of autophagy-related genes in host anti-Mtb immunity should be taken into consideration and need to be further characterized. On the other hand, it has been shown that the mycobacterial ESX-1 secretory system is required for activation of the host xenophagy pathway,^24,117^ which might support the supposition that Mtb prevents autophagosome–lysosome fusion at the late stage of infection.^78,79^ Furthermore, by monitoring autophagosome formation and subsequent degradation of autophagic cargo (a process termed autophagy flux) in infected cells, a research group found that virulent Mtb strains selectively prevented autophagosomes from fusing with lysosomes, while the autophagosomes that did not contain Mtb developed normally.^79,136^ These findings imply that Mtb has probably adapted to persist in autophagosomal vacuoles by inhibiting their degradation, which means it creates a sheltered environment for prolonged intracellular survival. Moreover, Mtb appears to selectively prevent xenophagic flux rather than the entirety of autophagic flux in host cells, which would likely result in hyperinflammatory responses and cell death.^137^ These hypotheses are supported by our finding that cytosolic Mtb can induce autophagy recognition and activation via a highly conserved ubiquitin-binding associated (UBA) domain on its surface to avoid excessive host inflammatory responses.^81^ Consistently, it has also been reported that, in a certain case, xenophagy can be beneficial for Mtb replication.^63^ Notably, the host autophagy pathway has been proposed as a potential target for host-directed anti-TB therapy,^138^ and based on these new concepts, a promising candidate of drugs or agents is expected to selectively target Mtb-containing autophagosomal vacuoles rather than cause nonselective overall interference in host autophagic flux. In addition, these drugs should not only enhance autophagy activation but also overcome the Mtb-induced blockade of autophagosome–lysosome fusion.

### Novel mechanisms by which Mtb targets innate immune regulatory machinery

**Fig. 3:**
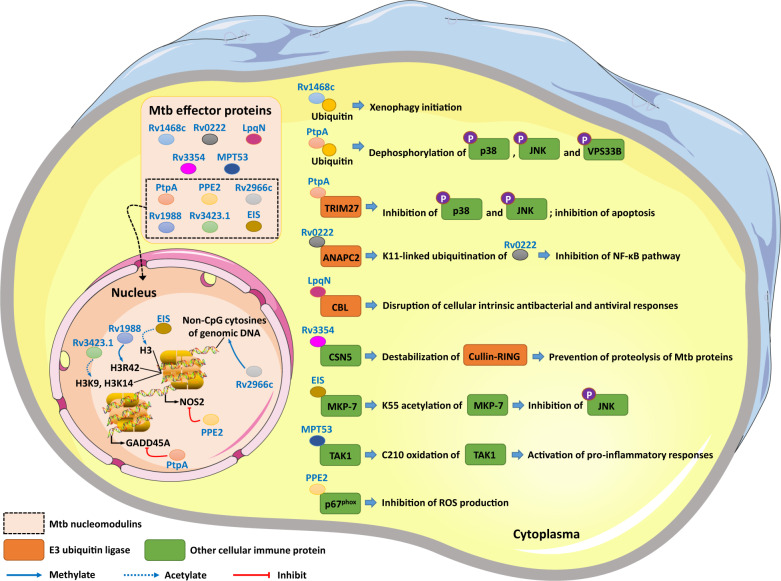
Mtb effector molecules targeting the host cellular and molecular regulatory machinery of innate and intrinsic immunity. Upon infection, Mtb delivers a wide range of effector proteins into host cells for direct interaction with the molecular components of the host ubiquitin system and other intrinsic cellular immune proteins, thus subverting host innate immunity. In parallel, some Mtb effectors are identified as nucleomodulins, which enter into the host nucleus and are involved in epigenetic and transcriptional regulation of host innate immune responses

The increase in the number of studies has tremendously expanded our understanding of multifaceted molecular mechanisms by which Mtb modulates the host immune regulatory network for its own advantage. In this section, we discuss the newly elucidated strategies adopted by Mtb to manipulate the host regulatory machinery of cellular innate and intrinsic immune responses via direct host–pathogen molecular interactions (Fig. 3).

### Mtb targeting of intranuclear immune regulatory machinery

Nucleus targeting has been emerging as a new aspect of the regulatory mechanism adopted by bacterial pathogens to manipulate host cell physiology and subvert immune defenses. In particular, an increasing number of bacterial effectors have been found to enter the infected cell nucleus to hijack host nuclear processes, and these nuclear attackers are named “nucleomodulins”.^139^ Bacterial nucleomodulins may mimic eukaryotic transforming factors, transcription factors, chromatin-regulatory factors or posttranslational modifiers, intervening in host gene transcription, chromatin reorganization, RNA processing or DNA replication and repair.^139^ Recent studies have identified several mycobacterial nucleomodulins that exert a range of intranuclear regulatory functions, which are described below.

First, some Mtb nucleomodulins function as histone-modifying enzymes to engage in epigenetic regulation of host immune responses. Histone modification probably plays an essential role in the regulation of host anti-Mtb immunity, since inhibition of histone deacetylases (HDACs) in human monocytes leads to attenuated host immune clearance of Mtb.^140,141^ In addition, suppression of HDACs decreases matrix metalloproteinase-1 and -3 in Mtb-infected macrophages, whose proteins drive TB lung immunopathology.^142^ Furthermore, histone methylation and acetylation are closely associated with BCG-induced host-trained immunity against Mtb.^143,144^ Pulmonary TB patients undergo obvious changes in histone modification in blood leukocytes;^145^ similarly, individuals with clinical resistance to Mtb infection (known as TB resisters) display an altered expression pattern of genes related to histone modification in blood monocytes.^140^ To date, three Mtb effectors that target and modify host histones have been identified: EIS, Rv1988, and Rv3423.1. As previously described, Mtb EIS increases the acetylation level of histone H3 to regulate host autophagy activation during infection.^125^ Mtb Rv1988 localizes to the host chromatin during infection, serving as a functional methyltransferase that dimethylates an arginine residue at H3R42 to repress a range of host genes involved in reactive oxygen species (ROS) production, such as *NOX1*, *NOX4,* and *NOXA1* and nitric oxide synthase 2 (*NOS2*).^146^ Although the significance of Rv1988 on Mtb pathogenesis has not been identified, the expression of Rv1988 in nonvirulent *M. smegmatis* markedly enhanced bacterial survival in infected mice.^146^ Mtb Rv3423.1 was isolated from the chromatin of Mtb-infected human macrophages where it displayed histone acetyltransferase activity and targeted host H3K9 and H3K14 (ref. ^147^). Similarly, recombinant *M. smegmatis* Rv3423.1 exhibited advanced intracellular survival in macrophages.^147^

Second, Mtb nucleomodulin Rv2966c was identified as a 5-methylcytosine-specific DNA methyltransferase that participates in the methylation of host genomic DNA primarily at non-CpG cytosines upon infection.^148^ However, the immunomodulatory role of Rv2966c in host–Mtb interactions has not been clarified. Despite limited knowledge of the pathogenic contribution of Mtb-induced host DNA methylation changes, in Mtb-infected macrophages, hypermethylation was predominantly observed on genes related to host immune responses, such as NLRP3 inflammasome activation and pro-inflammatory cytokine production.^149–151^ These characteristics have been consistently observed in blood monocytes isolated from TB patients.^152^ In addition, blood monocytes from BCG-vaccinated individuals also displayed a different DNA methylation pattern and advanced capacity for mycobacterial control, indicating the involvement of DNA methylation in host-trained immunity against Mtb.^153^

Third, some Mtb protein effectors exhibit dual regulatory functions that not only target host cytosolic components but also mimic eukaryotic transcription factors involved in host intranuclear processes. For instance, the Mtb secretory protein PPE2 was found to directly interact with the host cytosolic subunit of NADPH oxidase, p67^phox^, via an SH3-like domain to inhibit ROS production and favor intracellular survival of Mtb in macrophages.^154^ Intriguingly, PPE2 also contains a eukaryotic-like nuclear localization signal (NLS), by which it can be translocated into the host nucleus via the classical importin α/β pathway.^155^ Thereafter, PPE2 binds to the *NOS2* promoter and limits host ROS production.^155^ In another example, early studies have demonstrated that Mtb PtpA is delivered into the host cytosol, where it directly targets the vacuolar-H^+^-ATPase machinery to inhibit phagosome acidification and the NF-κB pathway to suppress host inflammatory immune responses.^67,156^ Moreover, we recently found that Mtb PtpA can also enter the nucleus of infected cells, where it binds to and modulates the expression of diverse host genes, such as *GADD45A*, to affect cell proliferation and migration.^157^

The host nucleus plays a central role in governing the all cellular activity, through which both genetic and epigenetic regulation of host immune responses to Mtb are driven.^8^ However, our understanding of the mechanistic and pathological implications of Mtb-hijacked intranuclear processes in the host remains limited. For example, it remains unclear how Mtb spatiotemporally regulates the intra- and extranuclear functions of these nucleus-translocated effectors. Furthermore, the majority of the identified nucleomodulins do not contain a classic NLS or nuclear export signal; what is the mechanism by which they shuttle between the host nucleus and cytosol? Further investigations are warranted to answer these questions and to verify whether a blockade of the host nucleus targeted by Mtb may be a new and valid approach of anti-TB treatment.

### Mtb targets the host ubiquitin system

The ubiquitin system refers to a network of proteins comprising enzymes that engage in ubiquitination and deubiquitination of cellular targets and ubiquitin receptors that decipher the ubiquitin code and translate it into cellular responses.^158^ This elaborate system regulates a wide range of cellular immune responses and plays a vital role in host–pathogen interactions.^159,160^ Upon infection with Mtb, host cells upregulate the E3 ubiquitin ligase-encoding genes *mkrn1* and *cops5* and downregulate *zfp91*, *ndfp2*, *ube2f*, *rnft1*, *psmb6,* and *psmd13*. Although the in vivo roles of these E3 ubiquitin ligases during Mtb infection have not been clarified, this finding suggests that Mtb likely interferes with cellular ubiquitination processes. Another early study confirmed this assumption by showing that Mtb-secreted virulence factor Rv3354 was able to interact with the metalloprotease (JAMM) domain of subunit 5 in the constitutive photomorphogenesis 9 signalosome (CSN5), resulting in the disruption of CSN5-mediated stabilization of cullin-really interesting new gene (RING) ubiquitin E3 enzymatic activity.^161^ Consistently, in our previous study, we provided direct evidence for Mtb targeting the host ubiquitin system by showing that Mtb PtpA directly binds to the RING domain of a host E3 ubiquitin ligase, tripartite motif containing 27 (TRIM27), to antagonize TRIM27-promoted inflammatory immune responses and cell apoptosis.^162^ Furthermore, it was recently reported that another Mtb-secreted virulence factor, LpqN, directly interacts with the human E3 ubiquitin ligase CBL, which plays a regulatory role in cell-intrinsic responses to infection.^163^ Intriguingly, mycobacteria possess pupylation, the covalent modification of protein lysine residues with a ubiquitin-like protein called Pup, but not ubiquitination as in eukaryotic cells.^164^ Our recent study noted that to efficiently interfere with host immunity, Mtb not only simply inhibits ubiquitin ligase-mediated immunomodulatory functions but also subtly exploits the host ubiquitin system for its own advantage. We found that, by direct interaction with ubiquitin via a unique ubiquitin-interacting motif-like region, Mtb PtpA is activated to dephosphorylate host p-JNK, p-p38, and p-VPS33B, leading to suppression of innate immune responses.^156^ Similarly, we verified that another Mtb ubiquitin-binding protein, Rv1468c, resides on the bacterial surface, as mentioned above, and it directly recruited host cytosolic ubiquitin to trigger xenophagy to restrict host inflammatory responses.^81^ More recently, we identified a Mtb-secreted protein effector, Rv0222, as a key suppressor of host NF-κB activation, showing that it undergoes K11-linked ubiquitination mediated by the host E3 ubiquitin ligase anaphase promoting complex (APC) subunit 2 (ANAPC2).^165^ Interestingly, rather than inducing the APC-mediated canonical ubiquitin-proteasome degradation pathway,^166^ K11-linked ubiquitination of Rv0222 facilitates the interaction between Src homology region 2 domain-containing phosphatase-1 and its adapter protein TRAF6, which blocks the K63-linked ubiquitination and activation of TRAF6, leading to inhibition of the NF-κB signaling pathway.^165^ In conclusion, targeting the host ubiquitin system is a recently emerging aspect of the tactics Mtb uses for immune evasion, which has received growing attention. Curiously, growing evidence suggests that the ubiquitin system is often coopted by invading pathogens and then plays an altered regulatory role in host immune responses. Future research will continuously expand our understanding of the ubiquitin system at the interface of host–Mtb interactions, particularly the undefined roles of host-originated and Mtb-mimicking E3 ubiquitin ligases, deubiquitinases, and ubiquitin receptors.

### Mtb targets intrinsic cellular immune components

Mtb has evolved to secrete a wide range of protein effectors via its sophisticated ESX secretion systems to counter host immunity.^124,167^ In particular, growing numbers of mycobacterial effectors have been linked to direct protein–protein interactions with the host to target and modify key cellular intrinsic antibacterial machinery. For example, it has been found that Mtb encodes eleven eukaryote-like serine-threonine protein kinases, including PknA to PknL (but not PknC), and two tyrosine phosphatases, PtpA and PtpB.^124^ Among these proteins, PknG is likely to selectively downregulate host PKC-α to inhibit the biogenesis of phagolysosomes.^168^ PtpA dephosphorylates host p-VPS33B, p-JNK, and p-p38 as described above, inhibiting phagosome acidification and the production of TNF and IL-1β in macrophages^67,156^; PtpB decreases the phosphorylation of host p65, IKKα, Erk1/2, and p38, suppressing macrophage apoptosis and the secretion of inflammatory cytokines.^169,170^ Both PtpA and PtpB are indispensable for Mtb intracellular survival.^156,171^ Although the host substrates of these Mtb eukaryotic-like kinases/phosphatases remain largely unknown, their essential roles in Mtb virulence have been well documented.^124,172^ Apart from phosphorylation-associated regulation of host factors, it was found that Mtb EIS can target and acetylate mitogen-activated protein kinase phosphatase-7 to prevent host JNK-dependent immune responses.^173^ In addition, in our recent work, we revealed an Mtb disulfide-bond-forming-like protein, MPT53, that can directly oxidize thiols on TAK1 to facilitate TAK1-mediated host hyperinflammatory immune responses.^174^

In contrast to the abovementioned cellular factors that control pathogen infection indirectly through the activation of signaling cascades followed by innate immune responses, some other host proteins are constitutively expressed in certain cell types and directly act to restrict pathogen growth, and they are termed “restriction factors”.^175^ Cellular restriction factors provide a frontline defense against invading microorganisms in a system known as host “intrinsic immunity”—a form of innate immunity initially elucidated in hosts as a mechanism to control viral infections. As discussed before, a range of antiviral immune mechanisms, such as cytosolic immune surveillance and the type I IFN response, are also involved in the host control of Mtb infection, indicating that the host might adopt certain shared cellular immune machinery, which may include the similar restriction factors, upon infection by viruses and bacteria. For example, IFN-induced transmembrane (IFITM) family proteins are well-characterized host antiviral restriction factors critical for controlling the entry and intracellular replication of viral pathogens,^176^ which has recently been associated with host anti-Mtb defense mechanisms as well. Specifically, IFTM1, IFTM2, and IFTM3 are required for the host restriction of Mtb intracellular growth in both human macrophages and lung alveolar cells, among which IFTM3 was shown to colocalize with Mtb phagosomes and contribute to phagosomal acidification.^177^ Another group of host intrinsic antiviral restriction factors, tripartite motif proteins (TRIMs), have also been demonstrated to engage in the host control of Mtb infection.^162,178,179^ In turn, these host restriction factors may be targeted by Mtb for immune evasion. As discussed above, Mtb LpqN is able to interact with host CBL, which is a restriction factor that regulates the balance between cellular intrinsic antibacterial and antiviral responses.^163^ Similarly, Mtb PtpA can directly bind host TRIM27 to antagonize its intrinsic immune functions.^162^ Furthermore, it was reported that TRIM14 is recruited to Mtb phagosomes in macrophages to act as a negative regulator of host cytosolic DNA-sensing pathway-dependent mycobacterial restriction.^180^ Together, these findings suggest potential strategies utilized by Mtb to avoid host intrinsic immunity.

Despite the compelling findings supporting an essential role for various cellular intrinsic protein factors in host anti-Mtb immunity, the determinant molecules of host resistance to TB infection remain largely unexplored. The application of recently developed research methods, such as genome-wide association analysis of human TB patients, may help to reveal the genetic etiology of TB and to identify key anti-Mtb intrinsic immune components.^181^ Furthermore, there is still a limited understanding of the direct interactions between Mtb-secreted proteins and host proteins, which play central roles in TB pathogenesis. Thus, more studies based on valid screening systems, such as the affinity tag purification mass spectrometry system, the MycoMarT7 transposon system, and the CRISPR–Cas9 screening system,^163,182,183^ are warranted to further improve our understanding of the Mtb–host network of molecular interactions.

## Conclusions

Our understanding of the interplay between Mtb and the host innate immune system has extensively expanded in recent years. As summarized in this review, upon Mtb infection, various cellular antimicrobial components respond to the activation of host innate immune surveillance pathways, which might be modulated by Mtb for its benefit. Moreover, an increasing number of studies have revealed emerging Mtb strategies to exploit the host molecular regulatory machinery of the innate immune system, including Mtb-mediated disruption of the host intranuclear immune regulatory machinery, the ubiquitin system and intrinsic cellular immune components. Thus, recent research on host–Mtb interactions has changed the traditional view that the pathogen is incompatible, and in conflict with its host until one is overwhelmed. As a particularly successful intracellular pathogen, Mtb has evolved much more moderate and nuanced strategies for immune modulation and evasion, with the principal aim of adapting to an intracellular niche for prolonged survival, rather than simply destroying the host. Therefore, it is not surprising that some mycobacterial factors have an inhibitory effect on host cellular antibacterial mechanisms (e.g., interfering with protective Th1-type cytokine production, vacuolar membrane trafficking, or autophagy activation), while others appear to play an opposite regulatory role. In fact, host immune responses are spatiotemporally regulated and dynamically changed throughout the course of TB.^184,185^ Therefore, Mtb probably tends to employ distinct effectors at different stages to bilaterally modulate the host immune machinery to establish a successful long-term infection. This concept is supported by compelling evidence indicating that, while an early protective Th1-type response favors a host-controlled infection, the machinery is often suppressed or exploited by Mtb, for example, to induce hyperinflammation at the late stage of infection, which causes lung cavitation and thus benefits bacterial transmission.^186^ Therefore, more in-depth studies are warranted to gain further insights into the regulatory mechanisms by which Mtb establishes innate immune evasion, providing knowledge that may help in the identification of either host- or pathogen-directed anti-TB therapeutic targets and contribute to the design of more efficient vaccines.
